# Evaluation of recurrent hyphema after trabeculectomy with ultrabiomicroscopy 50-80 MHz: a case report

**DOI:** 10.1186/1756-0500-5-549

**Published:** 2012-10-04

**Authors:** Giuseppe Mannino, Sara Verrilli, Silvia Calafiore, Angela Ciarnella, Alessandro Cutini, Cristina Mannino, Andrea Perdicchi, Santi Maria Recupero

**Affiliations:** 1 Ophthalmology Unit, NESMOS Department, S.Andrea Hospital, Faculty of Medicine and Psychology, University of Rome “Sapienza”, Via di Grottarossa, 1035 00189 , Rome, Italy

**Keywords:** Ultrabiomicroscopy, Recurrent hyphema, Trabeculectomy

## Abstract

**Background:**

Hyphema is a complication that can occur after glaucoma filtering surgery. Biomicroscopic examination of the anterior segment is commonly used to diagnose it and gonioscopy may provide a useful support to find the source of the haemorrhage. Unfortunately, when the blood hides the structure of the anterior segment the gonioscopic examination fails. In this case we performed ultrabiomiscroscopy with 50–80 MHz probes to overcome the limits of gonioscopy. The use of this technique to study the anterior segment of the eye has previously been reported in literature, but we illustrates its importance for performing a correct diagnosis in a specific case of hyphema.

**Case presentation:**

We report a case of a sixty-year-old caucasian male with recurrent hyphema in the left eye. The episodes of hyphema were four in two years and the patient came to the hospital for the first time in the last occasion. The past episodes were managed with topical corticosteroids and mydriatic drops. He referred surgical trabeculectomy in both eyes 5 years before the first symptoms and no specific eye trauma before the first episode. The examination of the anterior segment revealed a 2 mm hyphema in the left eye due to blood leakage through the superior iridectomy. Gonioscopy could not identify the source of the haemorrhage. B-scan ultrasound and ultrabiomiscroscopy, with 50–80 MHz probes, were performed. Ultrabiomiscroscopy, mainly with the probe of 80 MHz, provided images of high resolution of the structures of the anterior segment and it allowed the visualization of an abnormal vessel at the inner margin of the trabeculectomy opening, probably responsible of the recurrent hyphema.

**Conclusion:**

Ultrabiomicroscopy proved to be a useful diagnostic technique for identifying the cause of the recurrent hyphema when other examination techniques are not applicable.

## Background

Hyphema is a complication that can occur after glaucoma filtering surgery, although the causes are not always well known [1,2]. In some cases abnormal vessels have been detected at the internal margin of the trabeculectomy opening [3] and they are supposed to be the cause of the haemorrhage.

Gonioscopy normally, when present, can show the neovascularization of the irido-corneal angle but it has a limited application when blood hides the structures of the anterior segment.

In these cases ultrabiomicroscopy (UBM), using 50 MHz and 80 MHz probes, can provide images of high resolution of the anterior segment revealing the cause of the haemorrhage. We have reported a case of recurrent hyphema in a patient with an abnormal vessel at the internal margin of the trabeculectomy successfully found with UBM.

## Case presentation

**Figure 1:**
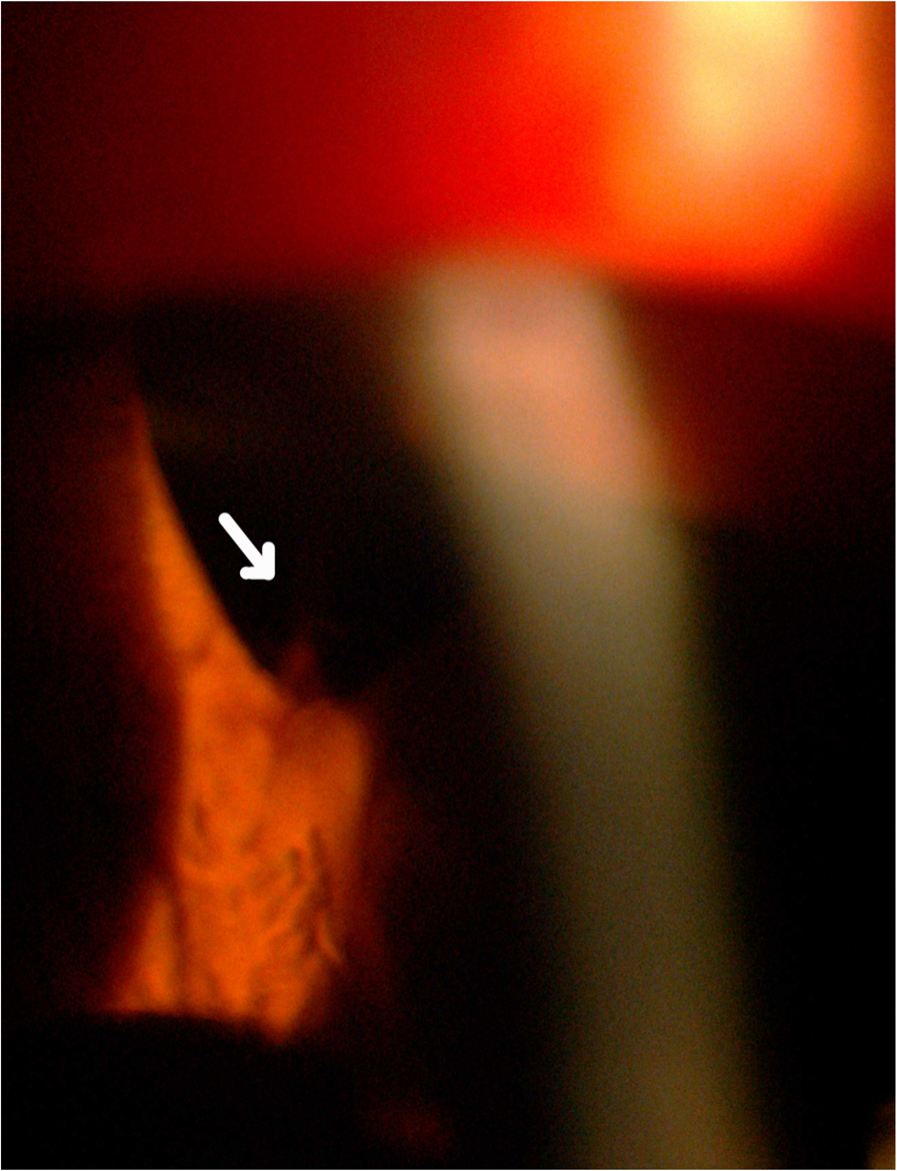
**Slit-lamp photo of the left eye.** Slit-lamp examination of the anterior segment revealing active bleeding in the anterior chamber. Blood coming out from the superior iridectomy (arrow).

**Figure 2:**
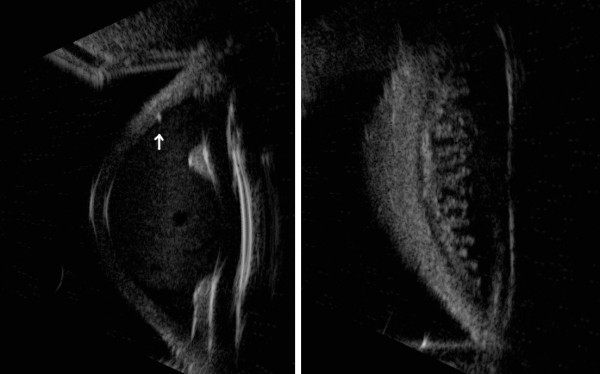
**Ultrabiomicroscopy 50 MHz of the involved eye.** Ultrabiomicroscopy with 50 MHz probe showing a hyperreflective area at the internal margin of the trabeculectomy (arrow) and the presence of corpuscolated elements in the anterior chamber. The posterior chamber seems to be within normal limits. The lens is correctly placed in the sulcus (on the left side). There are no signs of iris or ciliar processes haemorrhage (on the right side).

**Figure 3:**
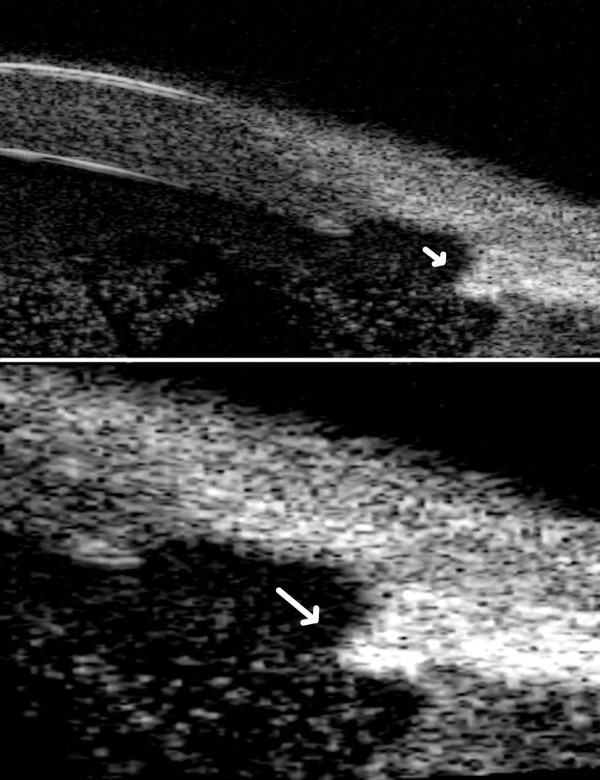
**Ultrabiomicroscopy 80 MHz of the involved eye.** Ultrabiomicroscopy with 80 MHz probe providing a more detailed image of the hyperreflective area found with 50 MHz probe. The 80 MHz probe reveals hyperreflective elements jutting into the anterior chamber from the inner margin of the trabeculectomy opening (arrow). On the lower part there is a magnified image of the trabeculectomy area. The features of the trabeculectomy can be well studied with this technique.

In July 2011, a 60-year-old caucasian male came to our Department referring recurrent episodes of hyphema in the left eye (LE). The patient reported no previous eye trauma, systemic hypertension or other systemic diseases and the bleedings were not associated with any physical efforts or drugs such as anticoagulants. He referred bilateral cataract extractions 7 years before, followed by trabeculectomy two years later in both eyes. At the moment of our observation the patient was under maximal topical medical therapy for glaucoma with timolol, apraclonidin, brinzolamide and acetazolamide because of the failure of filtering surgery. Best corrected visual acuity (BCVA) was 20/20 in the right eye and 20/40 in the left eye. At the slit-lamp examination the intraocular lens (IOL) was placed in the sulcus in both eyes and an active bleeding in the anterior chamber of the LE causing hyphema was present (Figure 1). The cataract surgery was done in another hospital and there was no information about the reasons of the position of the lens in the sulcus. In fact the posterior capsule was normal and there was no other alteration that can be related to surgical complications. The position of the haptics was studied with UBM and they seemed to be in the right place and far from iridectomy. Anterior segment in the right eye (RE) was normal. Biomicroscopic fundus examination showed vitreous detachment in the LE and negative findings in the RE. Intraocular pressure (IOP) measured with Goldmann tonometry was 14 mmHg in both eyes. A gonioscopic examination was done, but the blood present in the anterior chamber of the LE covered the irido-corneal angle structures. B-scan ocular ultrasound showed no pathological echoes in the vitreous chamber but the cause of the haemorrhage could not be identified. Ultrabiomiscropy was performed to study the area of the trabeculectomy, iris and ciliary processes. A 50 MHz probe was first used and it revealed an area of hyperreflectivity at the internal margin of the trabeculectomy with hyperreflective elements that moved from the anterior to the posterior chamber through the iridectomy and the pupillary hole in the LE. Iris and ciliary processes were normal with no sign of haemorrhage and the IOL was rightly placed in the sulcus (Figure 2). The UBM with 80 MHz probe confirmed the presence of a hyperreflective area at the inner margin of the trabeculectomy and it also showed hyperreflective elements jutting into the anterior chamber from the same area. This site was supposed to be the source of the haemorrhage (Figure 3) and an argon laser coagulation was performed on it without problems in the visualization of the abnormal vessel. The parameters of the laser treatment were 20 spots, 50 μm of diameters, 600 mW of power and 100 msec of time. Before doing laser a topical therapy with heparin drops, steroids and mydriatic was done for seven days without any result. After a 18-month follow-up the patient did not report any other episode of hyphema.

## Discussion

The aim of the therapy of glaucoma is to preserve the visual function and to avoid progressive anatomical and functional damages with minimal side effects [4]. The reduction of intraocular pressure is commonly obtained by the use of topical drugs as the first-line treatment of glaucoma. When this therapy fails, both laser and surgical therapies are useful in the management of glaucoma. Filtering trabeculectomy remains the “gold standard” therapy for long-lasting intraocular pressure reduction in uncontrolled glaucoma [5]. It works through a trans-scleral fistula that allows the outflow of the aqueous humour in the subconjunctival space, establishing a filtering bleb. The most known complications of trabeculectomy surgery are bleb leakage and failure, hyphema, retinal and choroidal haemorrhages, endophthalmitis, chronic hypotony, cataract [1,2].

Hyphema consists in the presence of blood in the anterior chamber of the eye. It may appear as a reddish tinge or it may appear as a small pool in the lower part of the anterior chamber. Hyphema is often caused by injury and according with its extension it may partially or completely block vision. Among the other causes of hyphema there are uveitis, intraocular tumours, congenital disorders, systemic hypertension, retinal detachment, haematological diseases and cataract surgery [6,7]. Hyphema can be rarely associated with antiplatelet or anticoagulant therapy [8] and also with Fuchs’ heterochromic cyclitis (FHC) [9]. In our case we also excluded the uveitis-glaucoma-hyphema (UGH) syndrome, which may occur after cataract surgery [10]. Moreover recurrent hyphema has been described as both an early and late complication of glaucoma filtering surgery [1,3]. Wilensky [3] hypothesized that after trabeculectomy the cases of recurrent hyphema are more frequent than those reported. This happens mainly in older patients who have a poor visual acuity because of the contemporary presence of cataract and glaucoma, in which slight changes of visual acuity might not be noticed. Most of hyphemas spontaneously resolve without complications but in some cases they may require a specific treatment. In fact a long-standing hyphema may result in hemosiderosis and heterochromia. Blood accumulation may also cause an increase of the intraocular pressure, which is dangerous mostly in glaucomatous eyes. In most of the cases of hyphema the interruption of any antiplatelet or antitrombotic drugs, the elevation of the head of approximately 45 degrees associated with bedrest are sufficient to its resolution. In refractory cases or in those with associated high intraocular pressure, argon laser treatment is useful to stop directly the source of the haemorrhage. The identification of the source of bleeding is crucial and UBM can be helpful to get it.

In this case report, the patient complained of recurrent hyphema after a failed glaucoma filtering surgery and it was decided to perform a laser treatment because of the recurrent episodes of hyphema. Slit-lamp examination revealed an active bleeding through the superior iridectomy but it was not capable of finding the source of the haemorrhage. B-scan ocular ultrasound with 10 MHz probe, did not show any pathological echoes in the vitreous chamber and according to literature, UBM with 50 and 80 MHz probes was carried out to explore the trabeculectomy area, the anterior and the posterior segments [11,12]. UBM with 50 MHz probe showed no active bleeding from iris or ciliar processes related to IOL and it revealed a hyperreflective area at the internal margin of the trabeculectomy. These features were better studied with UBM 80 MHz probe that showed a hyperreflective area from which hyperreflective elements jutted in the anterior chamber. These images likely corresponded to an anomalous bleeding vessel at the internal margin of the trabeculectomy and probably the hyperreflective elements were blood cells.

Comparing UBM using 80 MHz probe with the other above mentioned diagnostic techniques, we suggest the usefulness of its high resolution images for detecting the precise site of the haemorrhage on which a successful targeted argon laser coagulation was performed.

Moreover ultrabiomicroscopy may prove a suitable diagnostic technique in all those conditions in which detailed images of the eye structure are required. This is valid both for primary ocular diseases and for ocular involvement in systemic conditions.

## Conclusions

In our opinion, ultrabiomicroscopy proved to be a useful diagnostic technique for identifying the cause of the recurrent hyphema when other examination techniques are not applicable.

## Consent

Written informed consent was obtained from the patient for publication of this case report and any accompanying images. A copy of the written consent is available for review by the Editor-in-Chief of this journal.

## Competing interests

The authors declare that they have no competing interests.

## Authors’ contributions

SV and GM contributed to this case report’s conception and design. They also performed the literature research, prepared the manuscript and reviewed it for publication. CM, SC and AC were involved in the literature review and helped draft parts of the manuscript. SMR, AP and SV supervised the writing of the manuscript. SMR, AC and AP supervised the general management and follow-up of the patient. All authors have read and approved the final manuscript.
